# An outlook on structural biology after AlphaFold: tools, limits and perspectives

**DOI:** 10.1002/2211-5463.13902

**Published:** 2024-09-23

**Authors:** Serena Rosignoli, Maddalena Pacelli, Francesca Manganiello, Alessandro Paiardini

**Affiliations:** ^1^ Department of Biochemical sciences “A. Rossi Fanelli” Sapienza Università di Roma Italy

**Keywords:** AlphaFold, machine learning, structural bioinformatics, structure prediction

## Abstract

AlphaFold and similar groundbreaking, AI‐based tools, have revolutionized the field of structural bioinformatics, with their remarkable accuracy in *ab‐initio* protein structure prediction. This success has catalyzed the development of new software and pipelines aimed at incorporating AlphaFold's predictions, often focusing on addressing the algorithm's remaining challenges. Here, we present the current landscape of structural bioinformatics shaped by AlphaFold, and discuss how the field is dynamically responding to this revolution, with new software, methods, and pipelines. While the excitement around AI‐based tools led to their widespread application, it is essential to acknowledge that their practical success hinges on their integration into established protocols within structural bioinformatics, often neglected in the context of AI‐driven advancements. Indeed, user‐driven intervention is still as pivotal in the structure prediction process as in complementing state‐of‐the‐art algorithms with functional and biological knowledge.

## Graphical abstract

Rendering of a GFP‐like fluorescent protein, starting from its AF‐generated PDB coordinates. Like AI algorithms for image generation focus on creating realistic and detailed content, AI‐based algorithms for protein structure generation aim to accurately generate/predict complex molecular arrangements. Figure generated using CGDream (https://cgdream.ai).

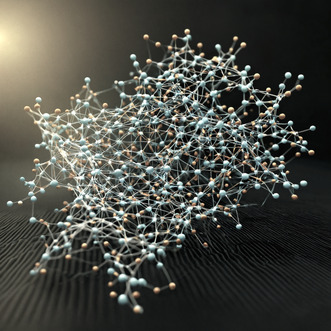

**Abbreviations**
AF: AlphaFold2
AF3: AlphaFold 3
AFDB: AF database
AI: artificial intelligence
CASP: critical assessment of protein structure prediction
EDAM: ontology of bioscientific data analysis and data management
ESM: evolutionary scale modeling
GDTTS: global distance test total score
MD: molecular dynamics
MSA: multiple sequence alignment
PDB: Protein Data Bank
plDDT: predicted local distance difference test
pMHC: peptide‐major histocompatibility complex
SCOP: structural classification of proteins
TCR: T‐cell receptor
TM: transmembrane proteins
UX/UI: user experience/user interface
XAI: explainable artificial intelligence

The advent of AlphaFold2 (AF) and its landmark performance at the 14th edition of the Critical Assessment of Protein Structure Prediction (CASP) marked a substantial shift in biomedical research, with the newfound ability to easily access millions of 3D‐structures of proteins, for which only their sequence was previously known [1, 2]. Within a year from AF's debut, a collaborative effort with EMBL‐EBI led to the creation of the UniProt‐indexed AF database (AFDB) [3, 4]. By releasing more than 200 million AF‐predicted structures, AFDB significantly enhanced the accessibility to this groundbreaking tool for the global research community. Overall, these advancements have significantly enhanced the structural coverage of the human proteome. Initially limited to just 10% when relying solely on experimental structures, this coverage has now expanded to 58% with the incorporation of high‐accuracy AF models (predicted Local Distance Difference Test, plDDT, scores above 70) [5, 6, 7]. However, this “Big Bang” of the protein structures universe, ignited by AF, also prompted critical examination regarding the accessibility, accuracy, reliability, and potential biases inherent in the data produced. Therefore, to avoid the potential risk of relying too much on readily available 3D‐structures without critical thinking and judgment, it is important to ask: “How do people from various academic backgrounds or research settings make use of this wealth of new structural information?”

## CASP—CAtalyzing a shift in the paradigm

**Fig. 1:**
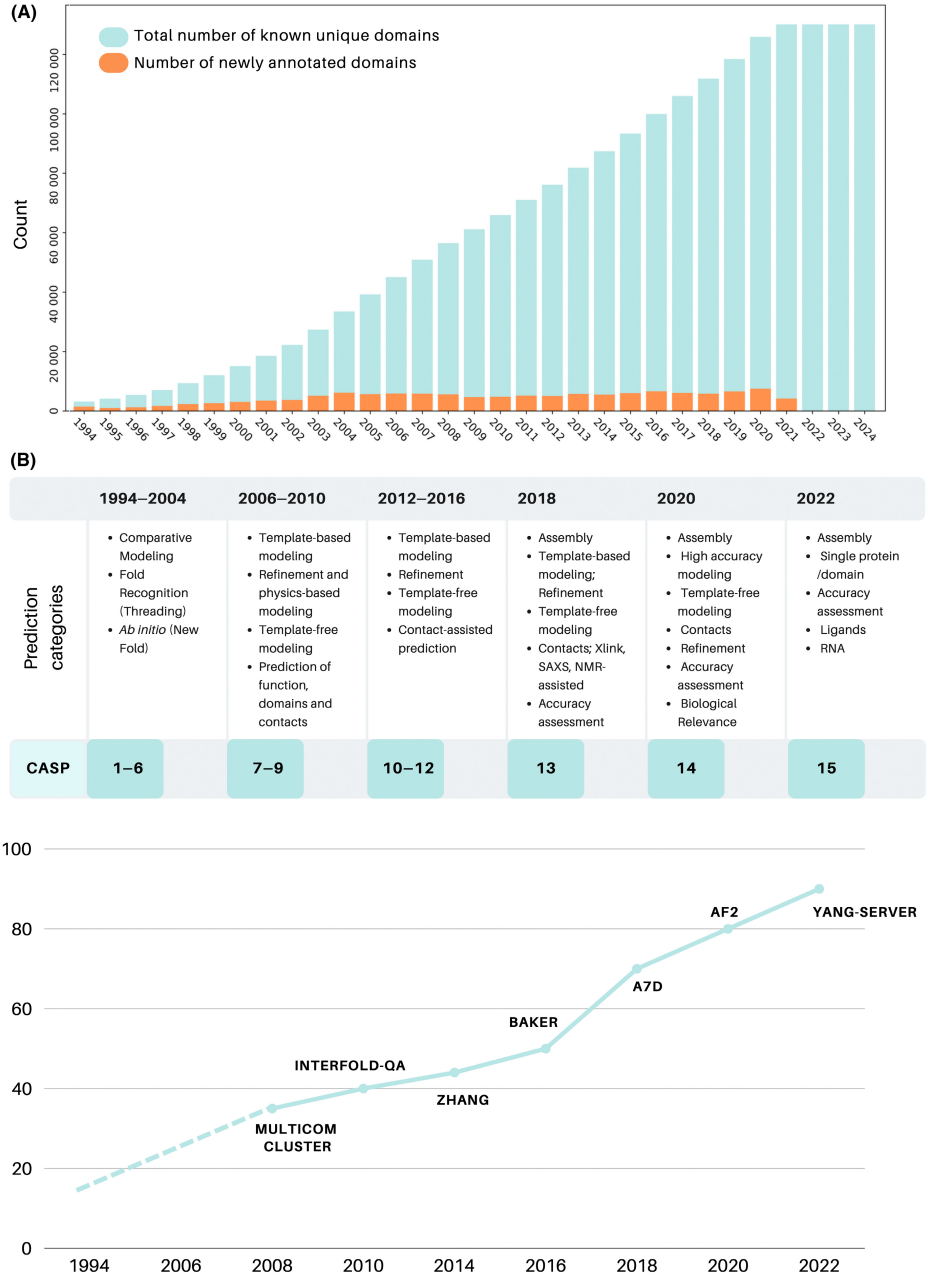
CASP: CAtalyzing a Shift in the Paradigm. In analyzing the events in the field of protein structure prediction, a shifting trend emerges in recent years according to various metrics. (A) The data from domain annotation in SCOP (Structural Classification of Proteins), as reported by the Protein Data Bank Statistics (RCSB PDB – Growth in Domain SCOP [8]), is plotted to show the number of known domains available each year from 1994 to 2024 (light blue). The orange highlights indicate the newly annotated domains each year. This visualization reveals, for the first time in 2022, an absence of newly annotated domains. This suggests that as experimental information on domain structures has increased, methods for accurately predicting structural domains have become more prominent. The table (B) reports on the prediction categories independently assessed at the Critical Assessment of protein Structure Prediction (CASP) over the years, mirroring the progress in the field and highlighting the most significant conceptual changes observed at CASP15. The plot illustrates the progression of the median GDTTS (Global Distance Test Total Score) values across different CASP editions from 2008 till now.

From its foundation in 1994, to the groundbreaking achievements at CASP14, the evolution of CASP mirrors the progress in Computational Biology and the growing intersection with Artificial Intelligence (AI) (Fig. 1) [9, 10]. In the first decade of the CASP competitions, the division into categories—Comparative Modeling, Fold Recognition, and *Ab‐Initio* Prediction—accompanied the birth of the first template‐based modeling algorithms [11, 12, 13], such as MODELLER [14], and algorithms for *ab‐Initio* folding, primarily governed by fragment‐based algorithms, notably Rosetta [15, 16] and I‐Tasser [17, 18].

The concept of coevolution, which is utilized in the AF algorithm, has been proposed since the early 1990s [19]. It suggests that detecting co‐evolutionary signals within multiple sequence alignments (MSAs) could indicate potential physical interactions between molecules. Clearly, even before the advancements of CASP14, leading‐edge methods already utilized the analysis of co‐evolutionary patterns through MSAs. Over the years, these methods have been refined to effectively distinguish between direct correlations, signifying actual physical contact, and indirect correlations. The latter may not denote direct interaction but still reveal a relationship, possibly due to other influencing factors [20, 21]. However, at that time, identifying accurate signals required extensive MSAs and computational resources, which were not readily available, leading to a diminished focus on this methodology.

The decade from 2010 to 2020 witnessed a significant shift as traditional bioinformatics began to integrate more effectively with advanced AI techniques. This period saw the resurgence of interest in the concept of co‐evolutionary signals [22, 23, 24], which were then combined with deep learning models to better predict protein contact patterns [25, 26]. This approach was based on the understanding that protein contacts are not randomly distributed, but they have a biological sense, given by domains and structural motifs of the category of proteins. Subsequently, in CASP 13, the use of the contact predictions concept was replaced in the first version of AlphaFold by neural‐network based distance probabilities [27], employing the steepest descent method as an optimization algorithm—a strategy that, although not yet fully successful, will later be crucial for enhancing accuracy. However, it was the revised neural network model proposed in CASP14 [1, 2], utilizing MSA transformers to extract co‐evolutionary signals directly from raw MSAs [28], which proved to be pivotal for enhancing accuracy.

After the milestone of CASP14, predicting single protein domains with accuracy has become significantly less challenging. Interestingly, this milestone coincides with an important accomplishment: the convergence of the domain structural knowledge (Fig. 1A). Throughout its history, CASP has dynamically evolved with a balance between retiring and modulating specific categories, while maintaining the core principles underlying the assessment process of the CASP competition largely unchanged (Fig. 1B). This evolution persisted up to CASP14, which saw significant changes, including the discontinuation of categories such as contact prediction and refinement for single protein models, alongside the introduction of novel challenges toward universal modeling [29, 30].

## Building on the outcomes of AlphaFold

Immediately after AF's performance at CASP14, and further propelled by the open‐source availability of the AF code, researchers in the field became aware of the vast potential inherent to the huge amount of available structural information and moved accordingly to ensure an optimal integration of AF into their specific areas of research. In this sense, a dual approach has been undertaken by the scientific community, mainly with the aim of addressing some of the limitations of AF, for example, the lack of physico‐chemical interpretation of proteins and their folding process. On the one hand, some efforts led to the development of platforms and tools that not only incorporate the algorithm's capabilities but also ensure they complement and enhance traditional physics‐based protocols (Table 1). On the other hand, a different set of initiatives has taken a more exploratory approach, capitalizing on the novel capabilities and insights provided by AF, and using them as a catalyst for expanding the application of AI to the biochemical and biological fields.

**Table 1:** Databases development and integrations after AlphaFold. The table organizes into categories the development of new databases and the integration of existing ones, following the release of the AlphaFold Database (AFDB).

Category	Tool	Description	Ref.
Development of new databases	AlphaFold Database	Initially comprising 360 000 predicted structures, the AFDB has expanded to over 214 million structures, providing a comprehensive resource for structural biologists	[3, 4]
	ESM Metagenomic Atlas	Offers structural predictions for 600 million metagenomic sequences, complementing the AFDB by extending coverage to environmental and microbiome samples	[31]
	AlphaFill	Enhances AF predictions by adding ligands from similar PDB structures, providing functional context to the predicted models	[32]
	TmAlphaFold	Incorporate predicted membrane planes into AF models, aiding in the study of membrane proteins	[33]
	AFTM	Leverages AF models to identify candidate human TMPs	[34]
Integration into software packages and existing data‐resources	CCP4 Suite	In crystallography, automatically fetch predicted structures from the AFDB to solve crystal structures by molecular replacement without user intervention	[35]
	MrBUMP and MrPARSE	Can automatically fetch AF predictions, integrating them seamlessly into existing crystallographic analysis pipelines	[36, 37]
	ISOLDE	It leverages AF predictions to refine models based on experimental data, such as cryo‐EM or X‐ray crystallography density maps	[38]
	CCP‐EM	Imports structures directly from the AFDB for electron microscopy applications	[39]
	ChimeraX	Uses ColabFold for modeling, retrieves structures from the AFDB, and provides interactive visualization of predicted aligned error (PAE) plots	[40]
	COOT	Imports AF models for detailed molecular modeling and refinement	[41]
	DALI Server and Foldseek Search Server	Perform structure‐based searches over the AFDB, enabling researchers to find structurally similar proteins	[42, 43]
	Jalview and Mol* Viewer	These tools import AF structures for sequence alignment and interactive 3D visualization, respectively	[44, 45]
	PHENIX	Integrates AF into molecular replacement pipelines, facilitating the incorporation of predicted structures into crystallographic workflows	[38, 46, 47]
	DeepTracer‐ID	Combines DeepTracer and AF to identify proteins in cryo‐EM maps by searching the AF library and iteratively refining the atomic model	[48]
	DeepProLigand	Uses DeepTracer and AF to predict protein‐ligand interactions by leveraging known structures available in the AlphaFold library or the RCSB PDB	[49]
	EMBUILD	Integrates U‐Net and AF to construct main chain maps and fit AlphaFold2 predicted chains into the maps for cryo‐EM applications	[50]

### AlphaFold integration in new databases

The establishment of a database derived from AF, that is AFDB, has significantly enhanced the efficiency of information sharing, emerging as an essential component for methodologies that depend on structural databases [51]. The AFDB contains predictions for all protein sequences annotated in the UniProt reference proteome, of length between 6 and 2700 amino acids, with the maximum limit decreasing to 1280 for noncurated sequences. A minimum length is required for having an informative MSA, while the maximum limits are set because of computational capacity. The integration of AFDB into pre‐existing databases represents a natural progression in response to AFDB's utility and relevance in the field. Indeed, key protein family databases such as InterPro and Pfam, as many others in the field [52, 53, 54, 55], have introduced dedicated pages for visualizing AF models. Additionally, the 2023 version of Ensembl [56] has incorporated AF models' visualization to map variant predictor effects onto the structure. Platforms such as PDB [8] and UniProt [57] are revolutionizing the management of predicted protein models within widely acknowledged databases, now incorporating AF predictions alongside experimental ones. The escalating volume of structures managed by these platforms has intensified the demand for scalability, a challenge efficiently met by renowned conformational search protocols, FoldSeek [42] and DALI [43], as well as newly developed ones [58]. Finally, new databases have been developed conveying newly available information to specific topics, for example, transmembrane proteins [33, 34]. It is important to note that this list is not exhaustive, and the landscape of AF integration is constantly evolving. Researchers are continuously finding new ways to incorporate AF into existing databases and cater to specific needs. These may include specialized databases for protein families, disease‐related proteins, or other specific fields. For instance, specialized protocols have emerged for analyzing kinases [59] and intrinsically disordered proteins [60]. The availability of such comprehensive data has significantly advanced high‐throughput and ‐omics research and has facilitated benchmarks of existing protocols, providing insights into their performance with predicted models [61, 62].

### AlphaFold for experimental structure determination

The advent of AF, with its remarkable accuracy in predicting protein structures, has ushered in a transformative era for experimental structural biology, opening new avenues for investigating complex biological systems. One of the most significant contributions of AF lies in its application to Molecular Replacement (MR) in X‐ray crystallography. Traditionally, MR has relied on experimentally determined structures from the PDB, posing limitations when suitable homologs were not available. However, AF has revolutionized MR by offering high‐quality predicted protein structures as alternative search models [63, 64]. This breakthrough has significantly expanded the scope of MR, making it applicable to a broader range of proteins, including those with no known homologs in the PDB [65]. The integration of AF predictions into established software [36, 37, 38, 46, 47] underscores its rapid adoption and widespread impact across various macromolecular structure determination methodologies. In PHENIX, AF models can be utilized through a dedicated set of functions [38, 46], including a PHENIX‐AF webservice to run predictions remotely from the GUI [47], their import from ColabFold [66], their trimming and splitting into single domains, and finally their positioning in unit cells. The resulting models can be examined with PHENIX validation tools to identify and manually fix any problematic areas. Similarly, CCP4 provides seamless integration of AF models for MR [35, 39, 41], interacting with the AFDB.

AF has been also integrated into various Cryo‐electron microscopy (cryo‐EM) pipelines, streamlining the workflow and improving both speed and accuracy [67]. Traditionally, building atomic models from cryo‐EM density maps has been a laborious and error‐prone process. AF predictions provide researchers with a high‐quality starting point. Software tools such as MrParse [36] and UCSF ChimeraX [40] can seamlessly access the AFDB, allowing researchers to achieve precise protein positioning within the cryo‐EM map by superimposing the AF model, which significantly improves the final model's quality, while substantially reducing manual building time. ISOLDE is another tool incorporating AF models during the refinement process [38]. This allows ISOLDE to utilize the predicted information alongside the cryo‐EM data, potentially leading to a more refined and accurate final structure in agreement with experimental data.

In particular, AF predictions can significantly improve the quality of cryo‐EM reconstructions, especially when dealing with data with low resolution and can be used as accurate starting models to fit components into cryo‐EM densities [67, 68, 69, 70, 71]. This is particularly helpful for determining the structures of large protein assemblies, such as the nucleopore complex [72]. Here, the authors utilized AF to enhance the structural determination of the nuclear pore complex's (NPC) cytoplasmic ring using integrative cryo‐EM. The high‐accuracy predictions of AF were crucial in providing detailed atomic models, accurately positioning proteins within the cryo‐EM density maps, and bridging gaps in incomplete experimental data. This integrative approach led to a more comprehensive and accurate model, revealing intricate protein interactions and conformations.

In another recent study, researchers working to solve the structure of the mycobacterial lipid transporter Mce1 were able to assign density to a previously unknown subunit of the complex, LucB protein. They were able to perform a structural search of a density‐derived poly‐Ala model against a large number of predictions in AFDB, which returned LucB as a hit. The assignment was subsequently experimentally validated [73].

### Unleashing the potential of AI in protein structure prediction

As the creation of AFDB streamlined the integration of AF into commonly utilized databases and tools [44, 45, 48, 49, 50], in a similar vein, releasing the source code of AF fostered the development of other AI‐based tools for protein structure prediction (Table 2). Consequently, AF promoted the idea that, as in other fields, AI was progressing exponentially, and promising results could also be achieved in structural biology. Although neural networks have long been applied to structural prediction, the point at which AI‐based predictions began to significantly outperform traditional methods coincided with the introduction of Transformer models [88], exemplified by the model used in AF.

**Table 2:** Deep‐learning‐based tools for protein structure and complex prediction. Summary of the deep‐learning‐based tools developed and/or improved after CASP14. The ‘Method’ column highlights the key distinguishing feature or unique aspect of each model. The aim is categorized as follows: MDM, multidomain modeling; PLC, protein–ligand complexes; PNC, protein–nucleic‐acids complexes; PPC, protein–protein complex; PSP, protein structure prediction.

Tool	Method	Release year	Aim	Ref.
AFSample	Stochastic perturbation of AF	2023	PPC	[74]
AlphaFold 3	Adapted AF + diffusion	2024	PPC; PNC; PLC	[75]
CombFold	AF + deterministic combinatorial assembly algorithm	2024	PPC	[76]
DeepAssembly	Population‐based evolutionary algorithm	2023	MDM	[77]
DMFold‐Multimer	DeepMSA	2024	PPC	[78]
EMBER3D	Protein language model	2022	PSP	[79]
EquiFold	SE(3)‐equivariant	2022	PSP	[80]
ESMFold	Protein language model	2023	PSP	[31]
HelixFold	Large‐scale protein language model	2023	PSP	[81]
MoLPC	AF + Monte Carlo tree search	2022	PPC	[82]
OmegaFold	Deep transformer‐based protein language model	2022	PSP	[83]
RosettaFold	Three‐track neural Network	2021	PSP	[84]
RosettaFold‐All‐Atom	Adapted RosettaFold + diffusion	2024	PPC; PNC; PLC	[85]
RosettaFoldNA	Adapted RosettaFold	2023	PNC	[86]
Umol	Evoformer + Structural module	2024	PLC	[87]

Almost in parallel with AF, the RosettaFold [84] neural network for protein structure prediction has emerged. The “two‐track” version of the network was outperforming trRosetta [2]. However, a performance improvement, approaching that of AF, has been observed with the development of a “three‐track” neural network. This latter was inspired by the features that contributed to the performance of AF, reworking them to operate in 3D coordinate space in order to establish a closer relationship between sequence, residue–residue distances and orientations, and atomic coordinates.

Methods such as AF, which rely on co‐evolution information extracted from MSA, inevitably hinder the possibility of prediction when an accurate MSA is lacking, as is the case of orphan proteins. The optimization and broader application of language models in different areas have paved a new direction in protein modeling too. These advancements have led to the development of MSA‐free approaches that are both computationally efficient and highly accurate. By leveraging the contextual understanding of protein sequences provided by language models, these tools can generate accurate predictions even in the absence of extensive sequence homology. Notable examples of these advancements include ESMFold [31], OmegaFold [83], and AminoBERT [89]. A significant achievement has been made with the large‐scale application of ESMFold, whose training data retrieval has been inspired by AF, culminating in the creation of their Metagenomic Atlas, comprising over 700 million predicted structures.

## Building on the limits of AlphaFold

With the AF exploit at CASP14 as a turning point, focus also shifted to exploring other essential aspects of structural prediction. This trajectory of progress transitioned into CASP15, which embraced “universal modeling” by expanding into RNA structure and protein–ligand complex prediction (Fig. 1B) [29]. CASP15 aimed to refine the evaluation metrics for RNA and protein–ligand complexes, underscoring the complexities in accurately predicting these structures.

Unfortunately, the outcomes of CASP15 fell short of expectations. In the case of RNA, none of the models presented managed to surpass the performance of methods evaluated in other competitions. For both RNA [90] and protein–ligand complexes [91], adaptations of algorithms typically applied to proteins were explored, but these adaptations achieved only limited success, potentially due to a lack of comprehensive training data. This suggests that, unlike with protein structure prediction, deep learning methods may find it challenging to leverage evolutionary data effectively in these specific areas. Regardless of whether the tools are directly inspired by the architecture of AF or push toward alternative methods, it is evident that the following AF unresolved issues are now central to the ongoing efforts in structural biology.

### Protein ligands and cofactors

The precise prediction of protein–ligand complexes plays a crucial role in overcoming various challenges in targeted therapies. As anticipated, after CASP14, several AI‐based methods for the universal modeling of proteins in combination with small molecules have been developed [87, 92]. While the performance of such methods is still not outperforming classical, physico‐chemical‐based approaches, it is expected that the parallel growth of both protein structure prediction and protein–ligand docking will lead to mutual benefits in the accurate prediction of protein–ligand complexes.

The inability to predict proteins in complex with ligands and cofactors, which is one of the main pinpointed limitations of AF, has been partially addressed by AlphaFill [32], which builds on AFDB. With this protocol, AF models can be enriched by tapping into the extensive resources of the PDB‐REDO [93] and CoFactor databases [94]. The protocol, validated for correlation in terms of root mean square deviation (RMSD) with the experimental structure both globally and locally, involves transplanting the most common ligands and cofactors from a sequence homologous to the AF model. This is achieved through a local structural superimposition of the model and the homologous experimental structure.

### Multidomain modeling

Homology modeling tools have emerged as an effective complement in scenarios where AF may not provide complete solutions. Structure prediction of GPCRs make up a paradigmatic case for the quality assessment of the predictions for a specific protein family, for which the structure determination has been critical so far. A comparative study involving AF, RoseTTAFold and MODELLER [95], confirmed the expected higher performance of MODELLER, whenever a high‐quality structural homolog is used as a template. Since this higher performance is evident when assessing interdomain positioning, a combined approach, utilizing both template‐based and template‐free methods, can yield effective results. Indeed, on this concept, recent pipelines have been developed to leverage the strengths of both approaches. One such example is AlphaMod [96], an automated pipeline that fuses AF with MODELLER, a well‐established template‐based modeling software. In a similar fashion, MoDAFold [97], combines AF with MD simulations to predict the structure of missense proteins with higher accuracy.

To address the problem of *ab‐initio* multidomain protein modeling, DeepAssembly, a new computational protocol for assembling multidomain proteins and complexes, was recently developed [77]. DeepAssembly uses a deep learning network to predict interdomain interactions, and then employs a population‐based evolutionary algorithm to assemble domains into complete structures. This approach outperforms AF in predicting interdomain distances in multidomain proteins and improves accuracy for low‐confidence structures in the AFDB.

### Protein–protein complexes

The “Assembly” category in CASP competitions has shown a notable upward trajectory since its introduction in CASP12 [98]. Even though it saw limited participation, it presented a significant opportunity for progress and quickly captured the interest of the scientific community, witnessing a surge in engagement in the subsequent years [99], and has now become one of the most hyped categories. Notably, Deepmind group did not take part in this competition, as their AF multimer version was not competitive enough [100]. However, building on the limits of AF, the quality of predictions within this category had improved substantially in CASP15 [101], with the first‐ranked DMFold‐Multimer [78], heavily influenced by the AF structural module. AlphaFold‐predicted pairwise subunit interactions can also be exploited for assembly prediction, as shown in new advancements that focused on MSA sampling, for example, AFsample and MULTICOM [74, 76, 102].

PROTAC modeling can be considered another pertinent example of the importance of protein complex prediction, even for nonphysiological interactions, in which AF fails to obtain accurate predictions [103]. Historically centered around a limited set of E3 ligases, the field is witnessing a shift with the discovery of new E3 ligase structures, opening avenues for their utilization and the rational design of ligands [104]. Yet, a notable gap remains in predicting the proper orientation of the E3–ligase complex concerning the target protein, which is essential for establishing a solid foundation for the design of ligands and linkers. Thus far, addressing such a challenge has involved utilizing a combination of tools for protein structure prediction, such as RosettaFold, along with protein docking techniques [105]. However the shift toward universal modeling is expected to provide a method to consider the orientation of the E3 Ligase–Target protein complex and the design of an appropriate bivalent ligand, at once.

### Protein–nucleic acids complexes

Understanding protein–nucleic acid interactions is particularly vital for decoding complex biological processes such as gene expression and genome repair, but accurate prediction presents significant computational hurdles due to the complexity of biomolecular interactions [106]. This knowledge is also pivotal for precision applications in genome editing, such as the engineering of Cas proteins, which are integral to technologies such as CRISPR‐Cas editing [107].

On the wave of universal modeling, the concepts and techniques underlying AF and RoseTTAFold have been extended to the prediction of the structures of nucleic acids and protein–nucleic acid complexes, leading to the development of AlphaFold3 (AF3) [75] and RosettaFoldNA [86], followed by RoseTTAFold All‐Atom [85]. Based on the RosettaFold three‐track neural networks for molecule representation, RoseTTAFold All‐Atom enhances this framework by incorporating atomic‐level details and chemical representations across various dimensions into a diffusion model. As input representation, the first track of RoseTTAFold All‐Atom encodes the sequence information of proteins and nucleic acids, including amino acid types and nucleotide bases. For nonpolymer atoms, it encodes their chemical element type. The second track represents pairwise information between atoms, including chemical bonds and distances. The last track includes the 3D coordinates of atoms or residues, along with information about chirality. The network of RoseTTAFold All‐Atom employs attention mechanisms to weigh the importance of different input features, allowing for dynamic and context‐dependent learning, and iteratively refines the predicted structure by updating the 3D coordinates based on the information from all three tracks. This integration significantly boosts the resolution and accuracy of the predicted molecular structures.

The AF3 architecture builds upon its predecessor by incorporating diffusion models as a generative module specifically designed for 3D structure generation [75]. Input data need additional preprocessing given the different molecular types the model has to handle. To do so, the raw inputs, the MSA, and the ligand conformers are converted into three different embeddings, namely the “Input,” “Pair,” and “Single” representations. The “Input” representation includes basic atomic and residue information such as type, position, and charge. The “Single” representation groups atoms by their amino acids or nucleotides, adding contextual information. The “Pair” representation captures spatial relationships in protein and DNA/RNA sequences, enriched with template and co‐evolutionary data. The “Pairformer,” which receives the enriched “Pair” representation, refines single and pair representations through recycling steps to produce a structural hypothesis, which then conditions the diffusion module for generating 3D coordinates. Despite the significance of AF's universal modeling generalization, the release of AF3 was notably different. The absence of source code and server constraints make it difficult to understand how AF3 generalizes and prevent its verification and replication.

Of note, such advancements boosted the research toward the application of deep learning methods also for predicting the secondary structures of DNA and RNA alone [108, 109, 110, 111].

### Protein dynamics

Exploring the dynamics of proteins has consistently presented a complex challenge, not just within the realm of computational predictions but also in experimental approaches. This challenge is linked to the prediction of protein complexes, as the interaction with other molecules often induces significant conformational changes in proteins [112]. The issue has gained renewed attention with the advent of AF, which, despite its advancements, tends to favor predictions biased toward more commonly represented conformations in its training data [113, 114]. In scenarios where conformational changes are subtle, implementing postprediction processing techniques and enhancing the accuracy of preliminary models (i.e., “refinement”) emerges as a viable strategy to mitigate this limitation. CASP10 assessment of the refinement category [115] has highlighted the effectiveness of refinement methods, especially those employing molecular dynamics, in producing conformations that in their highest accuracy find also suited application in MR.

In the era following CASP‐15, the endeavor to encompass the vast diversity of protein structural conformations can be expanded to most difficult and generalizable tasks. The approaches proposed leverage on tuning the MSA, that is, masking some positions, to guide the AF algorithm toward various conformational states [116, 117, 118, 119, 120]. The adoption of the flow‐matching method has significantly enhanced the accuracy of predicting protein conformational ensembles. This approach involves training generative models to closely replicate the distribution of protein conformations observed in experimental data or simulations [121, 122]. Another recent study showed that machine learning models can be trained on simulation data to directly create realistic protein structures without the need for extensive sampling, which significantly reduces computational cost [123]. The authors demonstrated this with a model called idpGAN, trained on coarse‐grained simulations of intrinsically disordered peptides. The model can predict new structures for sequences it has not seen before, proving its ability to generalize beyond the training data.

This evolution in strategy underscores the ongoing effort to refine computational tools for protein structure prediction, aiming for a more comprehensive and accurate representation of protein behavior.

### Orphan proteins

Methods such as AF, which rely on co‐evolution information extracted from Multiple Sequence Alignments (MSA), face challenges when predicting the structure of orphan proteins that lack accurate MSAs. This limitation has spurred the development of MSA‐free approaches that offer computationally efficient and highly accurate alternatives for predicting orphan protein structures. Notable examples of these advancements include ESM‐Fold [31] and OmegaFold [83], which leverage language models to bypass the limitations of MSA dependence. These models have shown promising results in predicting orphan protein structures, providing a valuable tool for understanding these previously enigmatic proteins.

A significant breakthrough in this field has been achieved with the large‐scale application of ESMFold, whose training data retrieval was inspired by AF. This effort culminated in the creation of their Metagenomic Atlas, a comprehensive repository of over 700 million predicted structures, including numerous orphan proteins. This atlas represents a major step forward in our understanding of the vast and diverse world of orphan proteins, offering valuable insights into their structures and potential functions.

Furthermore, the success of ESMFold and OmegaFold in predicting orphan protein structures has paved the way for further research and development in this area. Ongoing efforts are focused on refining these models, exploring novel MSA‐free approaches, and expanding the Metagenomic Atlas to include an even wider range of orphan proteins. Notable examples of these approaches are represented by HelixFold‐Single [81] and RGN [89]. The ultimate goal is to develop robust and reliable tools that can accurately predict the structures of all orphan proteins, unlocking their structural/functional peculiarities and contributing to our understanding of the complex biological processes they are involved in [124].

## Paths for the new era in structural biology

The advent of AF has ushered in a transformative era in structural biology, shifting the focus from merely predicting existing protein structures to the exploration and design of novel biomolecules. Indeed, while the interest in protein design and early successes can be dated back to several decades ago [125], with AI's unprecedented accuracy in predicting protein structures, researchers are now entering the era that the early work envisaged—where new proteins beyond the confines of known structures can be designed for practical applications and uses. This newfound capability opens exciting avenues, for example, engineering proteins with therapeutic potential, and crafting antibodies with enhanced specificity. The fusion of computational prediction and experimental validation is poised to revolutionize drug discovery, protein engineering, and synthetic biology, ultimately leading to the development of innovative therapeutics and biomaterials [126, 127, 128].

### *De novo* protein design

With 20 naturally occurring amino acids, a protein consisting of 100 amino acids could theoretically manifest in 20^100 different sequence variations. Given the diversity of protein sizes, the theoretical number of possible proteins far exceeds the number of proteins identified by nature [129]. *De novo* protein design leverages computational algorithms and biophysical principles to engineer novel proteins with tailored functions that, in billions of years of tinkering, Nature has never produced. Computational tools, often grounded in physics‐based energy functions and machine learning models, enable the exploration of vast sequence spaces and the prediction of protein structures. Recently, several protein design tools have been introduced by researchers (Table 3), which showcase substantial progress in the field. For a detailed review of *de novo* protein design, see Ref. [145]. Here, we will focus on two very recent and state‐of‐the‐art advancements in all‐atoms approaches, that is, RFdiffusion All‐Atom [85, 144] and ESM3 [135].

**Table 3:** Deep‐learning‐based tools for the *de novo* protein and peptide design. Summary of the deep‐learning‐based tools developed for protein/peptide design. The “Method” column highlights the key distinguishing feature or unique aspect of each model. The aim is categorized as follows: AD, antibody design; PD, protein design; PepD, peptide design.

Tool	Method	Release year	Aim	Ref.
ABlooper	Equivariant graph neural networks	2022	AD	[130]
Chroma	ChromaBackbone	2023	PD	[131]
DeepAb	Deep residual network	2022	AD	[132]
DeepH3	Deep residual network	2020	AD	[133]
EigenFold	Harmonic diffusion	2023	PD; PepD	[134]
ESM3	Generative language model	2024	PD; PepD	[135]
EvoDiff	Diffusion model	2023	PD	[136]
FoldingDiff	Transformer	2022	PD; PepD	[137]
FrameDiPT	SE(3) graph‐based diffusion model	2024	PD, PepD	[138]
GENIE	IPA, Evoformer	2022	PD	[139]
GRU‐based VAE	Variational autoencoders	2024	PepD	[140]
HelixDiff	Diffusion model	2024	PepD	[141]
HelixGAN	Generative adversarial network	2023	PepD	[142]
IgFold	AntiBERTy language model	2023	AD	[132]
MaSIF‐Seed	Geometric deep‐learning	2023	PepD	[143]
RFdiffusion	RosettaFold2	2023	PD; PepD	[144]

Recently, diffusion models have started to be used in protein and peptide design [146, 147]. These models, originally popularized in the field of generative art for their ability to create detailed and high‐fidelity images [148, 149], offer several advantages that make them suitable for protein modeling. Diffusion models work by iteratively refining an initial random structure into a coherent final one through a process that simulates the gradual “denoising” of a protein's atomic coordinates [150]. This iterative refinement allows for capturing the nuanced details of protein structures that are crucial for understanding their functions and interactions. While it is generally true that most neural networks are adept at capturing the global properties of protein structures [151], diffusion models offer an edge in generating high‐diversity folds, which in turn can be conditioned through a wide variety of inputs or design objectives [136]. In this field, the enhanced RFdiffusion All‐Atom model incorporates diverse biological building blocks such as DNA, RNA, ions, and small molecules, expanding the scope of protein design possibilities. When coupled to other tools such as ProteinMPNN [152], LigandMPNN [153], and AF (see, e.g., a design pipeline of heme‐binding proteins, available at: https://github.com/ikalvet/heme_binder_diffusion), it opens avenues for designing proteins with unprecedented sequences, structures, and functions. ESM3 [135] is another cutting‐edge AI model that can understand and design protein sequences, structures, and functions, using a frontier multimodal generative language model. The latter has been trained on a massive dataset of entries and can be prompted with any combination of sequence, structure, or function information to generate new proteins. Notably, it can generate proteins with characteristics not seen in nature, demonstrating its creativity in problem‐solving. As an example of its capabilities, ESM3 generated a new green fluorescent protein (esmGFP), which is significantly different from any known natural protein. This level of novelty is comparable to the amount of change that occurs in natural proteins over hundreds of millions of years of evolution. This demonstrates the potential of ESM3 as a powerful tool in protein engineering.

These techniques are revolutionizing protein engineering by enabling the rapid design of novel proteins with desired properties and, most importantly, have been experimentally validated, which in the end serves as the ultimate benchmark for the efficacy of predictive tools.

### Antibody design

Structure prediction of antibodies could be considered as a specialized area of protein structure prediction. In developing therapeutic antibodies, vaccines, and treatments for autoimmune disorders, the structural prediction of antibodies has historically depended on homology modeling, due to their highly evolutionarily conserved Y‐shaped scaffold [102]. However, predicting antibody structures, especially the highly variable complementary determining regions (CDR)‐H3 loop [154], remains challenging. Several methods have been developed to address this, utilizing both *ab initio* protocols and machine learning techniques. *Ab initio* protocols such as OptCDR [155], RosettaAntibody [156], and AbDesign [157] tackle this problem by redesigning CDRs to enhance antibody stability and affinity by optimizing conformational and free energy changes in specific residues. RosettaAntibody, for example, can perform *de novo* antibody design or affinity maturation of existing antibodies by classifying the antibody into regions, including the framework, canonical loops, and HCDR3 loop.

For more complex tasks, innovative antibody design protocols have emerged, including DeepH3, DeepAb, IgFold, and ABlooper, showcasing the power of machine learning techniques in antibody structural predictions [130, 132, 133, 158]. DeepH3, a deep residual neural network, identifies near‐native CDR‐H3 loops and improves the average RMSD of prediction compared to the standard Rosetta energy function. AbLooper employs Equivariant Graph Neural Networks to predict CDR structures, producing accurate antibody models efficiently.

Furthermore, pretrained language models have proven effective in inferring full atomic‐level protein structures. DeepAb leverages an antibody pretrained language model with recurrent neural network to reconstruct the entire antibody variable region, generating more precise structures compared with alternatives. IgFold, inspired by DeepAb, utilizes a pretrained language model trained on natural antibody sequences and graph networks to directly predict backbone atom coordinates, offering high speed, accuracy, and nanobody modeling capabilities.

While the remarkable advancements in domain prediction contribute to highly accurate models of immunoglobulin domains or target epitopes, the precise orientation and interaction between the CDR loops and the target epitope are areas that require further refinement [159, 160, 161]. These aspects of antibody–antigen interaction are more likely to benefit from the latest breakthroughs in predicting multimeric complexes, highlighting a crucial direction for future advancements in antibody design [130, 162, 163]. Sculptor is a new algorithm that addresses this challenge using deep generative design to create antibodies that bind to specific epitopes [164]. It does this by jointly searching for the best positions, interactions, and shapes of the protein scaffold. It then designs a protein backbone that complements the target.

In summary, while accurately predicting antibody structures, especially CDR‐H3, remains a challenge, significant progress has been made through the development of various computational methods and the integration of machine learning techniques and pretrained language models. These advancements hold promise for accelerating antibody design and engineering efforts, ultimately contributing to the development of more effective therapeutic antibodies.

### Peptide design

The development of therapeutic peptides hinges on the ability to design peptidic binders that target specific proteins of interest. Traditionally, peptide design has relied on a combination of rational design, simulation, and screening techniques [165]. Similarly, early AI‐based approaches to peptide design, which were adapted from protein design methods, employed various techniques such as inverse design (e.g., ProteinMPNN [152]), peptide‐specific methods (e.g., PepMLM [166]), and generative models (e.g., MaSIF‐Seed [143]). These approaches all aimed to design new peptide binders starting from the target protein. By leveraging different architectures, it is now possible to focus on the *de‐novo* design of peptide sequences. These approaches are particularly valuable for their ability to capture the distribution of amino acids that confer a set of functionalities and activities, such as antimicrobial, anticancer, immunogenic properties, or signal peptide functions. Variational autoencoders (VAEs), generative adversarial networks (GANs), and diffusion models have emerged as viable options. As an example, a recent multistep sequence generation algorithm was proposed [140]. The deep learning‐based generative model Gated Recurrent Unit based variational autoencoder (GRU‐based VAE) and the Metropolis Hasting (MH) sampling algorithm efficiently generate new peptide sequences. The binding affinity of generated peptides is then evaluated using physics‐based methods, such as molecular dynamics (MD) simulations. Several GANs have been trained for peptide design, tailored to specific use cases such as immunogenic, antimicrobial [167, 168, 169], and antiviral peptides [170]. Other examples of GANs, such as HelixGAN [142], have been specifically trained to focus on the design of helical peptides, and similarly, a diffusion model called HelixDiff [141] has been developed with the same objective. In a recent work [137], a diffusion‐based model (FoldingDiff) generating high‐quality backbone peptides (up to 128 residues) via a procedure inspired by the natural folding process, is presented. FoldingDiff uses a sequence of dihedral angles capturing the relative orientation of the constituent backbone atoms and generates stable folded peptides by denoising from an unfolded structure. Moreover, the development of combined and fine‐tuned approaches is becoming increasingly common. Latent space diffusion models, in particular, are gaining traction, and AMP‐Diffusion [171] exemplifies their application by harnessing the power of latent representations and the flexibility of diffusion processes to enhance the generation of antimicrobial peptides.

## Accessible software and hardware in protein structure prediction

With the widespread adoption and success of AI and computer science techniques in biology, it has become crucial to enable access to such prediction tools and technologies for a broad audience with limited familiarity with bioinformatics and software engineering. Ensuring easy access of AF and related tools to researchers from diverse backgrounds can permit new and diverse complex biological questions to be asked, and a fruitful *connubium* of AI and human expertise to be reached. Lowering such barriers and identifying the pivotal key factors essential for ensuring the success of a software release for scientists, clinicians, and students alike revolves around accessibility, user‐friendliness, and comprehensibility [172, 173]. For example, factors such as Graphical User Interfaces (GUIs) significantly reduce the barrier to software use, especially when coupled with User Experience/User Interface (UX/UI) studies and tutorials covering all aspects of user interaction [174, 175].

**Fig. 2:**
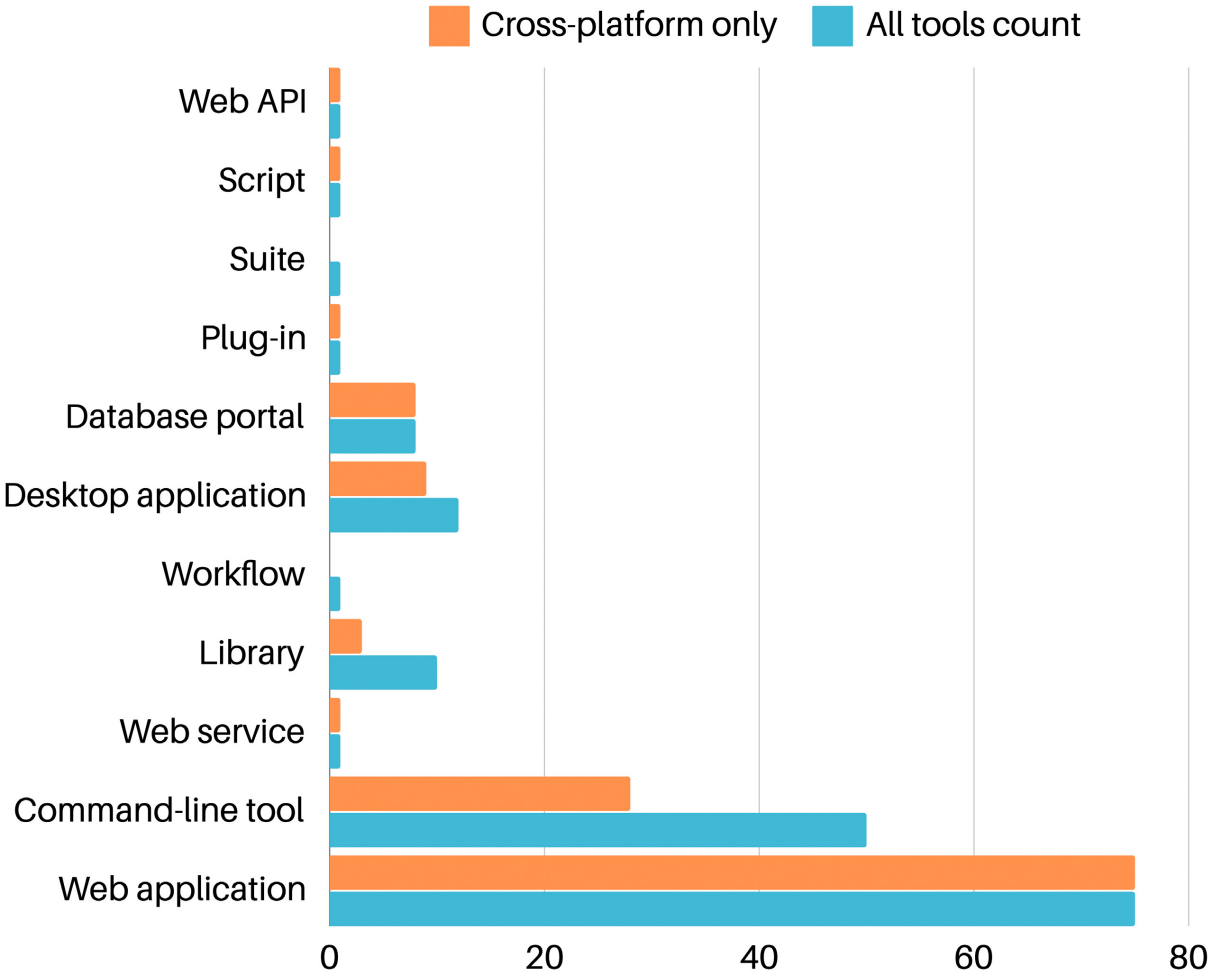
Overview of tools categorized under the “Structure Analysis” EDAM (Ontology of bioscientific Data Analysis and Management) “topic” tag in the Bio.Tools database; ([81], accessed on February, 2024). For each tool type, the plot displays the number of occurrences, along with the count of tools that are cross‐platform.

Analyzing the features of the other tools developed in the realm of protein structure prediction [176], the distribution of tool types reveals a strong inclination toward Web Application (Fig. 2). These are noted for their accessibility, yet they come with drawbacks such as server‐side dependence and limited control. Command‐Line Tools also feature prominently, showcasing their utility for batch processing. It emerges that Desktop Applications, which would offer unmatched control and independence from server constraints, are very limited as they face significant hurdles in cross‐platform compatibility. Widely used molecular graphics viewers constitute an exception. Indeed, the integration of AF has been promptly pursued in tools such as ChimeraX [40].

An example of a free interface for AF is seen with ColabFold [66] that, other than featuring an easy‐to‐use Colab notebook, implemented a faster MSA step and a way to customize the MSA. Delving into structure prediction interfaces, we can find some widely used web servers, that is, Phyre2 [177] and SwissModel [178], along with some Desktop Applications, like PyMod [179].

Now that AI models have become predominant in this field, the issue of accessibility is no longer solely related to the concept of GUIs and similar interfaces. The “black box” factor also comes into play, referring to the inability to explain what occurs during the process. Therefore, there is a growing need to develop Explainable AI (XAI) solutions that provide transparent insights into the decision‐making processes of these models, fostering trust, accountability, and understanding among users. Even if some attempts are ongoing [180], transparent AI solutions still need some time to become predominant in this domain.

While there is little room for improvement in increasing the modularity of environments that implement algorithms akin to AF, or similar “black box” models, there is potential in harnessing software development that facilitates a unified application of structural bioinformatics protocols, fostering a user‐driven methodology. However, there is a trend toward developing “blind” software—applications that take inputs and produce outputs without requiring users to understand the underlying processes. While this approach promotes efficiency and caters to users of different expertise levels, it risks discouraging deep understanding of the methodological principles. In structural bioinformatics, this may lead to insufficient appreciation of physico‐chemical principles such as protein folding and interactions, essential for accurate interpretation of results.

When examining the process of predicting 3D structures and its various stages, it becomes clear that an insufficient understanding and interaction between the user and the algorithm often results in suboptimal predictions [181]. A key aspect of this process is the selection of a structural template. This decision should be predominantly influenced by the user's expertise and judgment, rather than relying solely on sequence identity percentage metrics. Factors other than sequence similarity are functional characteristics, unique structural motifs or conformations, solvent composition, pH values, and the interaction with additional binding entities.

Another fundamental yet often overlooked and nonoptimized step is the quality of the MSA prior to the modeling step. A well‐built MSA can effectively link sequence information with the structural elements of the proteins, increasing the accuracy of the final model. As a remark of the importance of such a step, still in the post‐AF era, there are witnesses of effort in developing tools for facilitating the manipulation of multiple sequence alignments [182]. Manipulation here refers to refining the alignment, correcting gaps, and mis‐alignments, to ensure that it accurately reflects the evolutionary and functional relationships among the sequences. This consideration becomes particularly pertinent when addressing processes such as the prediction of multidomain and/or multimeric structures. As highlighted earlier, a significant challenge persists in realizing *ab‐initio* predictions of protein complexes. In addressing this challenge, a fusion of *ab‐initio* and homology modeling protocols, approached with a user‐driven perspective, can be a potential alternative by leveraging the possibility to integrate a variety of information sources.

In order to translate these objectives into reality, the figure of the Research Software Engineer (RSE) surges as central, a hybrid professional embodying the confluence of software engineering and scientific inquiry [183].

As software may become more accessible, computational resources must do so too. Protein structure prediction and design are recognized as Nondeterministic Polynomial‐hard problems [184], necessitating exponential computational efforts with traditional techniques. High‐Performance Computing (HPC) significantly influences this field, as many prediction algorithms benefit from parallelization across HPC's multiple processors [185]. Similarly, the rapid advancement in Graphical Processing Units (GPUs) enables efficient execution of these complex tasks, especially with deep learning algorithms [186]. Consequently, adapting existing tools for both parallel and GPU computing has become widespread.

## Conclusions and future perspectives

As extensively discussed in previous papers [187, 188, 189, 190, 191, 192, 193], the arrival of AF has fundamentally transformed our approach to structural biology. In this review, we have focused on the aspects most significantly influenced by the release of AF, examining limits and new opportunities, changes from the methodological perspectives, some state‐of‐the‐art applications of particular interest, and software development viewpoints. Until 2021, efforts have primarily been directed toward accurately predicting naturally occurring proteins, the imminent solution to this issue now shifts focus toward a variety of distinctly different domains: protein design, synthetic biology, AI‐driven drugs and antibodies design, integrative structural biology.

Each of these areas has had to adapt to the sudden availability of advanced structural information, now made readily accessible to a broad audience. This accessibility has not only democratized the field but also spurred a wave of innovation, necessitating a reevaluation of existing practices and the development of completely new methodologies to fully leverage the potential of this groundbreaking tool. A promising future direction is to integrate AI with quantum computing frameworks [185]. The development of hybrid quantum‐classical solvers, such as QPacker in Rosetta software [186], exemplifies this innovative approach, reshaping our understanding of complex energy landscapes in protein structures.

## Conflict of interest

The authors declare no conflict of interest.

## Author contributions

SR and MP collected, analyzed and interpreted the data, and wrote the paper. FM collected the data. AP wrote the paper, supervised, and conceived the project.
